# Retraction: Chronically high level of *tgfb1a* induction causes both hepatocellular carcinoma and cholangiocarcinoma via a dominant Erk pathway in zebrafish

**DOI:** 10.18632/oncotarget.28570

**Published:** 2024-05-14

**Authors:** Chuan Yan, Qiqi Yang, Han-Ming Shen, Jan M. Spitsbergen, Zhiyuan Gong

**Affiliations:** ^1^Department of Biological Sciences, National University of Singapore, Singapore; ^2^National University of Singapore Graduate School for Integrative Sciences and Engineering, National University of Singapore, Singapore; ^3^Department of Physiology, Yong Loo Lin School of Medicine, National University of Singapore, Singapore; ^4^Department of Microbiology, Oregon State University, Corvallis, OR, USA

**This article has been retracted:** Multiple figure images in this paper were found to contain internal overlaps and duplications. Specifically, duplicate images were discovered in Figures 1B, 4A, 4B and 5C. The authors were unable to make corrections using the original data. Therefore, with the agreement of all authors, the Scientific Integrity office at Oncotarget has decided to retract this paper.

Original article: Oncotarget. 2017; 8:77096–77109. 77096-77109. https://doi.org/10.18632/oncotarget.20357

